# The complete chloroplast genome sequences of the *Sicyos angulatus* (Cucurbitaceae)

**DOI:** 10.1080/23802359.2022.2093672

**Published:** 2022-07-08

**Authors:** Tae-Young Choi, Eun Su Kang, Dong Chan Son, Soo-Rang Lee

**Affiliations:** a Department of Biology Education, Chosun University, Gwangju, Republic of Korea; b Division of Forest Biodiversity, Korea National Arboretum, Pocheon, Republic of Korea

**Keywords:** *Sicyos angulatus*, complete chloroplast genome, Cucurbitaceae

## Abstract

*Sicyos angulatus* (burcucumber) is an annual plant native to the north-eastern America. We investigated the genomic characteristics of the complete chloroplast (CP) genome in *S. angulatus* with a de novo strategy. The CP genome was 154,986 bp in length including 84 protein coding genes, 37 tRNA genes, and eight rRNA genes. It has large single-copy (LSC) (84,355 bp), small single-copy (SSC) (18,079 bp), and a pair of inverted repeats (IRs) (26,276 bp), which consists of typical quadripartite structure. A phylogenetic analysis of 64 CP genomes from Cucurbitaceae revealed that the *Sicyos angulatus* was separated from other species and clustered together with *Sicyos edulis*, which is congruent with previous studies. Infrafamilial classification system inferred from our data was also congruent with previous study based on CP DNA data.

*Sicyos angulatus* Linnaeus 1753 (burcucumber) is an annual plant native to the north-eastern America. It has spread and can be found in humid region like waterside or floodplains from Florida, and west to Texas (Britton and Brown 1913). *Sicyos angulatus* is known as a noxious invasive plant and is spread throughout Europe and Asia (Osawa et al. 2016). Indeed, there have been reports about this invasive species in parts of South Korea, India, and several country in Asia (Lee et al. 2015; Thakur 2016). The high invasiveness is likely derived from its competitive ability of crowding out the neighboring plants (Zhao et al. 2019). Its vines can pull a crop to the ground, making crop harvesting nearly impossible, so it is problem to corn and soybean farmers (Esbenshade et al. 2001; Gibson et al. 2005). The genus *Sicyos* comprises approximately 40 species. It is one of the most diverse genera within the family Cucurbitaceae (Kobayashi et al. 2012). Some *Cucurbitaceae* species have been studied for its chloroplast (CP) genome characteristics (Zhang et al. 2006). However, the genomic information applicable for *S. angulatus* still remains absent. In this study, we investigated the genomic architecture of the whole CP genome for *S. angulatus* using whole genome shotgun sequencing.

We collected young leaves of *S. angulatus* from Buan-gun, South Korea (N 35°36′45.4″, E 126°17′05.8″). All material used for the study were collected legally. As all sites were not prohibited from sampling, permits were not required. The voucher specimen was prepared and deposited at the Herbarium of Chosun University (CHO; ra1130@chosun.ac.kr) with the accession number CHO0000132. The total genomic DNA was extracted followed by manufacturer’s protocol (Qiagen, Hilden, Germany). After library preparation, the prepared libraries were sequenced on Illumina HiSeq-X platform (Illumina, San Diego, CA). Sixty-three million high-quality 150 bp paired-end reads were obtained. We assembled 9.58 GB reads with *de novo* strategy using Geneious Prime (ver. 2021.2.2) followed by Gibbs (2019). The genes were predicted with GeSeq (Tillich et al. 2017), and manually curated based on Blast search result. The simple sequence repeats were investigated with MISA (Beier et al. 2017).

The complete CP genome of *S. angulatus* has been submitted to GenBank (accession no. OK514744). It is 154,986 bp in length with the typical quadripartite structure comprising a large single-copy (LSC) (84,355bp), a small single-copy (SSC) (18,079 bp), and a pair of inverted repeats (IRs) (26,276 bp). The CP genome contained 129 genes including 84 protein coding genes, 37 tRNA genes, and eight rRNA genes. Fifty-two simple sequence repeats were identified in the cp genome, which consist of 50 mono-nucleotide and two di-nucleotide.

To investigate its phylogenetic relationship, the concatenated CDs sequences from whole CP genome of 64 Cucurbitaceae and one outgroup taxa were aligned in MAFFT v. 7.450 (Katoh et al. 2019). All sequences except *S. angulatus* were downloaded from NCBI GenBank. Genus *Begonia* was selected as an outgroup following phylogenetic relationships based on a previous study (Zhang et al. 2006). We inferred the phylogeny using maximum-likelihood (ML) algorithm implemented in RAxML v. 8.2.11 with GTR GAMMA model. For the clade support, 1000 bootstrap replicates were used. The Sicyoeae species formed a monophyletic group (BP = 100) with strong support on ML tree (Figure 1). Although there were minor differences in tree topology, the overall groupings were congruent with previous study (Renner and Schaefer 2016; Figure 1).

**Figure 1.**
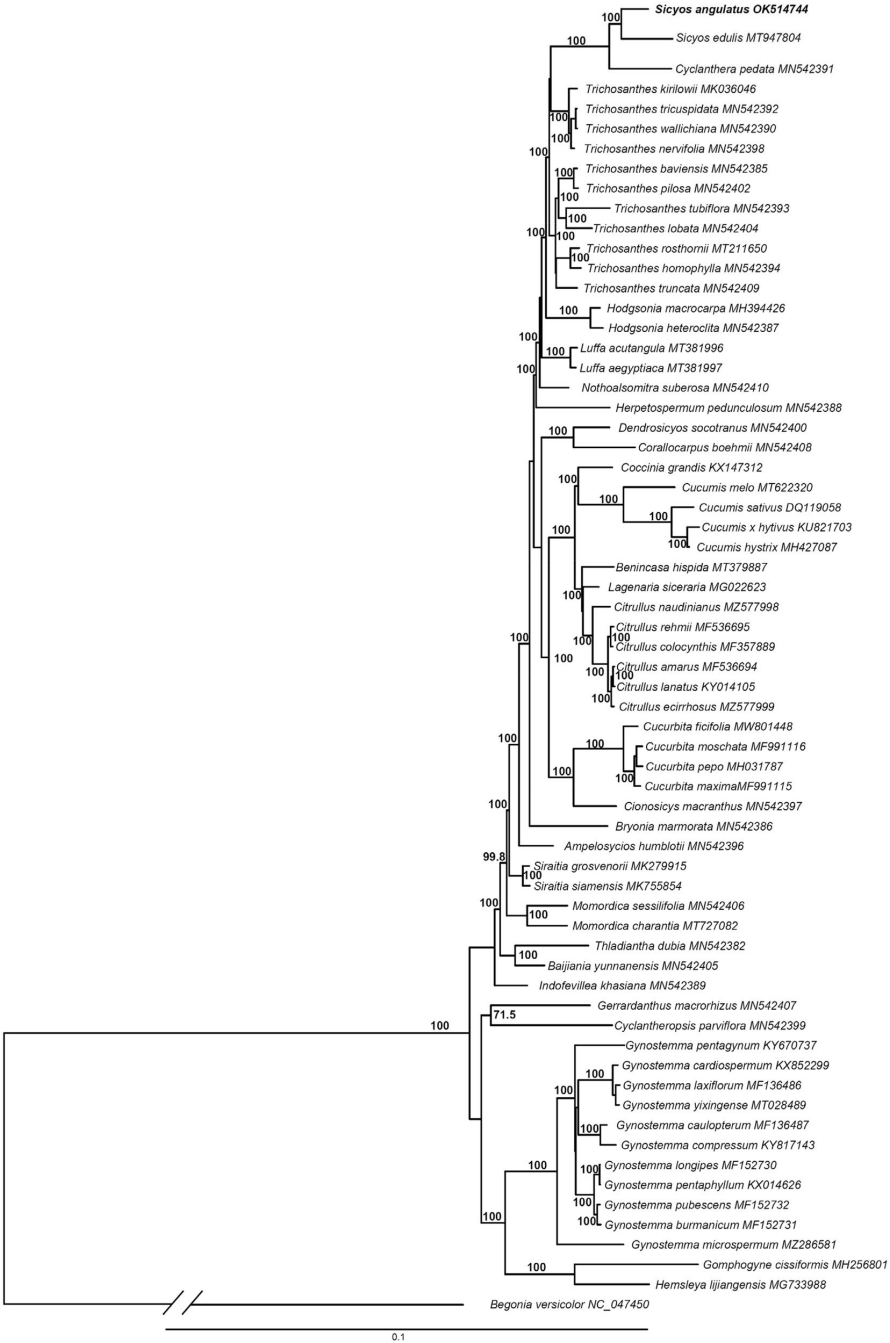
Maximum-likelihood (ML) tree based on chloroplast genome sequences of 65 species of Cucurbitaceae.

## Author contributions

DC Son and SR Lee conceived the research idea and designed the experiments. SR Lee got the funding and revised the manuscript. TY Choi and ES Kang conducted laboratory works and analysis. TY Choi and SR Lee wrote the manuscript. All authors revised and approved the final manuscript. All authors agree to be accountable for all aspects of the work.

## Data Availability

The genome sequence data that support the findings of this study are openly available in GenBank of NCBI at https://www.ncbi.nlm.nih.gov/ under the accession no. OK514744. The associated BioProject, SRA, and Bio-Sample numbers are PRJNA768979, SRP340194, and SAMN22073017, respectively.
